# Continuous assessment of cowpea [*Vigna unguiculata* L. Walp.] nutritional status using diagnosis and recommendation integrated system approach

**DOI:** 10.1038/s41598-023-40146-0

**Published:** 2023-09-02

**Authors:** Firmin Nonhouégnon Anago, Emile Codjo Agbangba, Gustave Dieudonné Dagbenonbakin, Lucien Guillaume Amadji

**Affiliations:** 1 Laboratory of Soil Sciences, School of Plant Production, Faculty of Agricultural Sciences, University of Abomey-Calavi, 01 BP 526 RP, Cotonou, Benin; 2 Laboratoire de Biomathématiques et d’Estimations Forestières, Faculté des Sciences Agronomiques, Université d’Abomey Calavi, 04 BP 1525 RP, Cotonou, Benin; 3 Laboratoire de Recherche en Biologie Appliquée, Département de Génie de l’Environnement, Ecole Polytechnique d’Abomey-Calavi, Université d’Abomey-Calavi, 01 BP 2009 RP, Cotonou, Benin; 4 Laboratory of Soil Science, Water and Environment, Research Agricultural Center of Agonkanmey, National Institute of Agronomic Research of Benin, 01 BP 988 RP, Cotonou, Benin

**Keywords:** Plant sciences, Environmental sciences

## Abstract

Cowpea is one of the widely cultivated and consumed grain legumes in Africa, but its production is hampered by soil fertility degradation on farms. Here, we assessed the spatial nutritional diagnosis of cowpea and the variability of their productivity using the diagnosis and recommendation integrated system (DRIS) and geostatistics tool. We achieved a sampling of 200 geo-referred points in cowpea farms in four communes of Benin. In addition, we determined grain yield and the content of N, P, K, Ca, Mg, and Zn in the leaves. From DRIS, the order of nutrient deficiency was as follows: P > K > Ca > Zn > N > Mg; P > K > Ca > N > Zn > Mg; N > Mg > Zn > K > P > Ca; P > Ca > K > N > Mg > Zn, at Dassa-Zoume, Glazoue, Ketou, and Ouesse, respectively. Sampling points were close enough to detect the spatial variability of the DRIS Index, mean of nutrient balance index (NBIm), and cowpea productivity (spatial dependence index ˃ 50%). The combined analysis of the cowpea relative yield and NBIm maps showed that the NBIm map effectively indicated the spatial distribution of cowpea productivity. The spatial variability of the DRIS index has provided an accurate guide to where adjustments to fertilization rates are needed.

## Introduction

Agricultural productivity is increasingly low due to progressive soil degradation and nutrient depletion^1,2^. Main soil nutrients (N, P, and K) as well as secondary and micro-nutrients are removed through harvesting, leaching, denitrification, erosion, and run-off. Soil fertility degradation adversely affects the food production systems in many African countries, causing the loss of topsoil which results in huge yield losses of important crops including cowpea^3^. Cowpea is one of the major green food crops that contribute to food security and poverty reduction worldwide. It is one of the most cultivated and consumed legumes in Africa. Cowpea leaves contain a greater proportion of protein than dry seeds, thus they are eaten and can be a substitute for animal protein in areas where leaves are not primarily used as fodder. Cowpea leaves and grains contain on average 27–43% and 21–33% crude protein^4^. It is also used as livestock fodder in West Africa^5^ and contributes to soil fertility improvement through symbiotic nitrogen fixation and ground cover. Unfortunately, cowpea productivity is affected by soil fertility degradation in Africa, especially in Benin^2^. In Benin, on-farm yield of cowpea is low and rarely exceeds 0.5 t/ha^6^. Therefore, effective management of soil fertility under cowpea production is very critical to ensure food and nutrition security in Benin and Africa at large.

The basis for effective soil fertility management is the application of the required amounts of nutrients according to soil type, crop, season, etc. Soil testing provides information on nutrient availability, which forms the basis for fertilizer recommendations to maximize crop yields^7^. The Basic assumption of the soil analysis method is that the chemical substances induce the absorption of soil nutrients by the root system. Therefore, soil portion explored by root, soil moisture, soil temperature, and aeration, and even the higher or lower absorption due to the own plant’s nutritional needs is not taken into account as factors in this method^8^. As soil fertility is its capacity to provide adequate nutrients for specified plants when other factors are favorable, tissue analysis is considered a more direct method of soil fertility evaluation than soil analysis^9–11^. Diagnosis and Recommendation Integrated System (DRIS) has emerged as one of the most accurate methods in detecting nutritional deficiencies and excesses as it accounts for the relationship among nutrients^11,12^. DRIS is a bivariate approach developed by Beaufils^13^ to interpret the results of leaf tissue analysis. This method processes the nutrient ratio to eliminate the influence of sampling time, plant growth stage, and leaf tissue position in the interpretation of leaf tissue analysis results regarding individual nutrient levels^11–13,23^. It is a tool to simultaneously identify nutrient deficiencies and excesses and their ranking.

Recent studies showed the effectiveness of DRIS in the nutritional status assessment of rubber^14^, oil palm^15^, tomato^16^, beech^16^, carrot^17^, garlic^18^, sugarcane^19^, banana^20^, guava^21^, maize^22^, yam^23^, soybean^24^. However, these studies interpreted plant nutritional status on basis of an average of nutrient indexes giving nutrient status for the whole studied area. DRIS fails then to give a continuous spatial nutrient status for plants. The integration of geostatistical tools into DRIS will enable to assess the continuous spatial variability of nutrient status. Ribeiro et al.^25^ observed that spatial variability of the DRIS Index efficiently indicated the points at which fittings in the fertilization doses are required. In addition, da Silva et al.^11^ reported that the use of a geostatistics tool resulted in a better understanding of the relationship between nutritional and non-nutritional variables on the Conilon coffee yield. Likewise, several studies reported that geostatistics is an efficient method for studying the geospatial dissemination of soil properties^1,26,27^. Therefore, the combined use of the geostatistic tool and DRIS will help describe precisely cowpea crop nutritional status and its spatial variability as well as the relationship between nutritional status and cowpea yield. This study aimed to assess the spatial nutritional diagnosis of cowpea crops and the spatial variability of their productivity.

## Material and methods

### Study Area and leaf sampling

**Figure 1:**
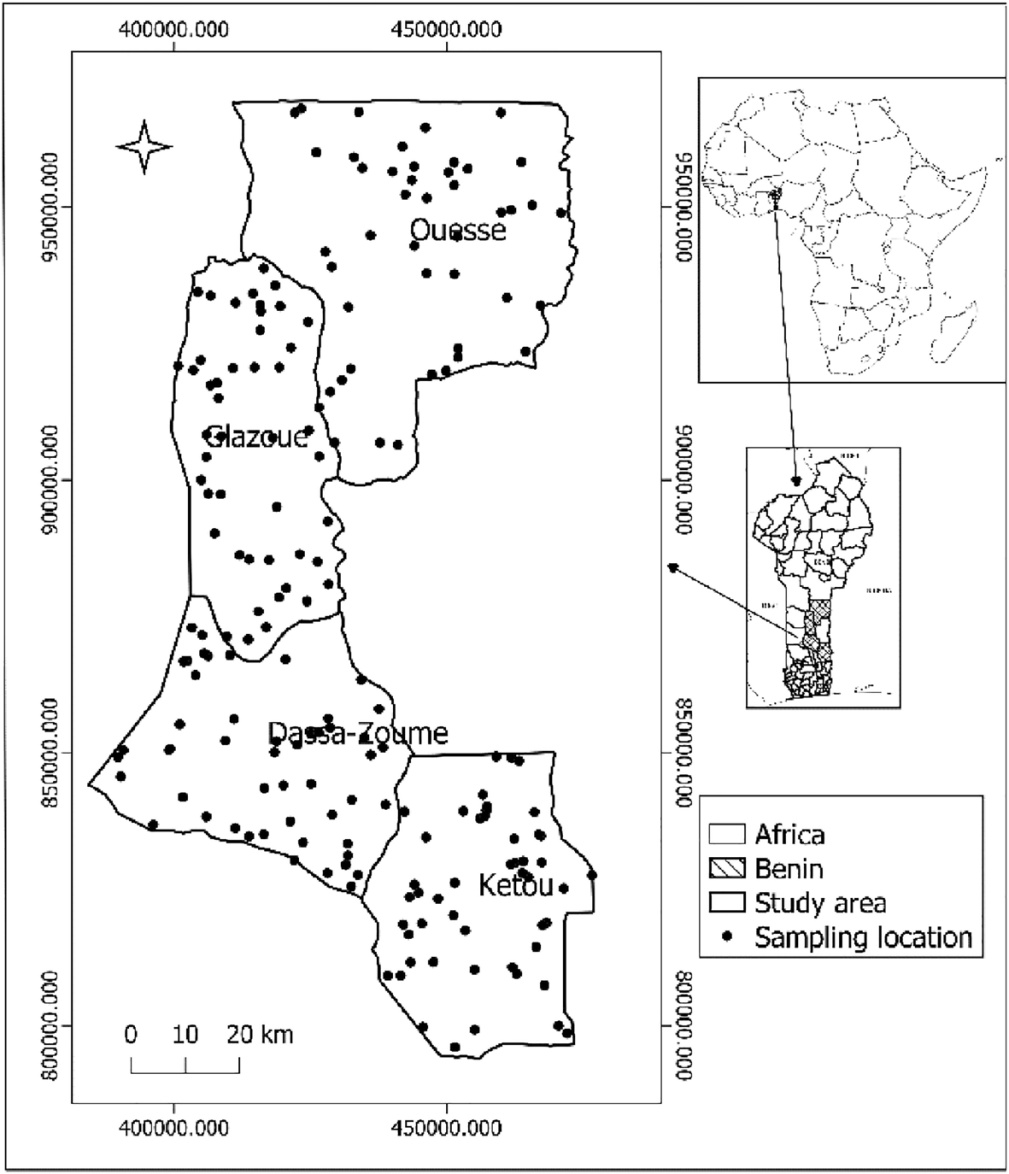
Map of the sampling area (detail for the sampling points).

Voucher specimens of *Vigna unguiculata* (L) Walp in Benin were identified and deposited at the Benin National Herbarium in 2001 by Adjakidjè^28^. The leaf sampling area consisted of farmer fields from the municipalities of Ouesse, Glazoue, Dassa-Zoume, and Ketou. Located in the center of Benin (1°5′–2°5′ of longitude E and 7°3′–8°5′ of latitude N) (Fig. 1), this zone was selected because it is the main area of cowpea cultivation in Benin ^2^. The predominant climate in the region is Sudano-Guinean with an average annual rainfall of about 800–1100 mm and the dominant soils (USDA system) are Ferric and Plintic Luvisol^29,30^.

In the study area, leaves were sampled from fields sowed with native cowpea seeds without fertilizer application during raining seasons of 2018–2019. The sampling area was randomly established and consisted of 200 geo-referred points (Fig. 1), each point representing a farmer’s field. Farmer cowpea field close to the geo-referred point is sampled in cases where the points were not in cowpea fields. These farmer fields were marked with a GPS device (Garmin Etrex Vista). In each farmer field, elementary sampling was done at the beginning of flowering and consisted of three fully developed leaves from the top of 30 cowpea plants^31^. Ninety (90) leaves were sampled per field and mixed to form a composite sample. This study conforms with the IUCN Policy Statement on Research Involving Species at Risk of Extinction and the Convention on Trade in Endangered Species of Wild Fauna and Flora.

### Yield estimation and leaf nutrients content

On each of the previously described farmer fields, four randomly selected 1 × 1 m^2^ plots were staked after sampling to determine the sowing density and to estimate cowpea yield. Plots were harvested and cowpea total aboveground biomass were weighed using a hand-held scale with 0.01 g of readability. Aboveground biomass samples were taken to estimate productivity. The harvest and leaf samples were oven dried at 65ºC until constant weight for dry matter estimation. Cowpea grain weight was calculated at 12% moisture content^32^. After drying, the leaf samples were milled in a mill-type Willey and passed through a sieve of 20 mm diameter. After milling, the powders were digested with a mixture of concentrated H_2_SO_4_ and H_2_O_2_^33^. N concentrations were determined by the Kjeldahl method with UDK 169 Automatic Kjeldahl analyzer from VELP Scientific^34^. K, Ca, Mg, and Zn concentrations were determined using Atomic Absorption Spectrophotometer method with Agilent 200 Series AA^35^. P concentration was determined using vanado–molybdate method with 1100 spectrophotometer from Fisher Bioblock Scientific^36^.

### DRIS norms and guidelines

Original data of nutrient contents in leaf samples were converted into g/kg, to allow comparison between nutrients from different units. Besides, leaf nutrient levels below the mean level reduced by 2.55 × standard deviation (SD) and above the mean level increased by 2.55 × SD were excluded. Therefore, nutrient levels that were within the mean values ± 2.55 × SD of the normal distribution (95% of data around the mean) were considered valid and the others were not included in all the procedures below^37^.

The first step in the use of DRIS to assess plant nutritional status is the establishment of DRIS norms^12^. Thus, the leaf nutrient composition data was divided into two subpopulations using the mean of cowpea grain yield + SD as the criteria for cut-off^22^. We then obtained two subpopulations, the high-yielding subpopulation in which the cowpea yields greater than or equal to 976.82 kg/ha, and the low-yielding subpopulation with grain yields lower than 976.82 kg/ha. The mean, SD, and coefficient of variation (CV) of the dual ratio between nutrients (N/P, P/N, N/K, K/N, etc.) were calculated in the two subpopulations. The variance ratio between subpopulations for all forms of expressions was calculated. Among different forms of the ratio between two nutrients, the one showing a higher variance ratio (variance of low yielding subpopulation/variance of high yielding subpopulation) was selected in high yielding subpopulation for establishing DRIS functions involved in the calculation of indexes^38–40^.

The DRIS indexes were calculated for all leaf nutrients contents assessed. The formula simplified by Jones^38^ was applied to calculate the DRIS functions for nutrient ratios, as follows:

1 $$f\left( \frac{A}{B} \right) = \left( {\frac{A}{B} - \frac{a}{b}} \right)/SD$$

where $\frac{A}{B}$ is nutrient ratios in each leaf sample; $\frac{a}{b}$ and $SD$ are mean ratios and standard deviation of the ratios of the desired population (high-yielding subpopulation).

After defining the functions DRIS, the DRIS index was calculated for each nutrient. The DRIS index was determined as follows:

2 $$DRIS\;index\;of\;A = \frac{{\sum f\left( \frac{A}{B} \right) - \sum f\left( \frac{B}{A} \right)}}{n}$$

where n is the number of DRIS functions of each dual ratio defined by criteria chosen of the norms, in that the A nutrient is involved; $f\left(\frac{A}{B}\right)$ is the functions for nutrient ratios, in that nutrient A appears in the numerator and $f\left(\frac{B}{A}\right)$ is the functions for nutrient ratios, in that nutrient A appears in the denominator.

The average nutritional balance index (NBIm) was obtained from the DRIS index as follows:

3 $$NBIm = { }\frac{1}{ni}{*}\left( {\left| {{\text{N}}\;{\text{index}}} \right| + {\text{ |P}}\;{\text{index}}\left| {{ } + { }} \right|{\text{K}}\;{\text{index}}\left| + \right|{\text{Ca}}\;{\text{index}}\left| { + { }} \right|{\text{Mg}}\;{\text{index}}\left| { + { }} \right|{\text{Z}}\;{\text{index}}|} \right)$$

where ni is the number of DRIS indexes involved in the analysis.

To classify cowpea’s nutritional status, the concept of potential response to fertilization^41^ was adopted. Therefore, three nutritional classes: deficient, suitable, and excessive were established as follows:

- Deficient when DRIS index < 0 and |DRIS index |> NBIm;
- Suitable when | DRIS index |< NBIm;
- Excessive when DRIS index > 0 and | DRIS index |> NBIm.

### Statistical and geostatistical analyses

Leaf nutrient contents, DRIS index, NBIm, and yield data were characterized using descriptive statistics (mean, SD, CV, and frequency). Shapiro–Wilk test was used to perform a normality test of DRIS ratios. In addition, the Pearson correlation was used to evaluate the correlation between the NBIm and the relative yield.

4 $${\text{relative}}\;{\text{yield}} = \frac{Observed\;yield}{{Potential\,yield}}*100$$

Geostatistical analysis of relative yield, NBIm, and DRIS Indexes was performed to quantify the spatial dependence, starting from the theoretical semivariograms. In addition, spherical, gaussian, exponential, and linear models were tested as experimental models. The model with the highest coefficient of determination (R^2^); and the highest spatial dependence index was chosen for each variable involved in this geostatistical analysis. Then, the values of relative yield, NBIm, and DRIS Indexes were estimated by the ordinary kriging. The spatial distribution mapping of relative yield, NBIm, and DRIS Indexes was performed using QGIS software.

### Permission to collect *Vigna unguiculata* L. Walp

In this study, the cowpea (*Vigna unguiculata* L. Walp**)** leaf samples were collected with the permission of the National Institute of Agricultural Research of Benin.

## Results

### Establishment of DRIS model parameters for cowpea

Of the 192 farmer cowpea fields prospected in Ketou, Dassa-Zoume, Glazoue, and Ouesse, 93 exhibited high cowpea grain yield (yield > 976.82 kg/ha) (Table 1). Cowpea grain yield in the high-yielding population was approximately 36% higher than in the low-yielding population (Table 1). The 15 ratios of nutrients selected from the high-yielding population to compose DRIS standards, showed a normal distribution and their coefficient of variation (CV) was less than 20% (Table 2).

**Table 1:** Mean and standard deviation (SD) of nutrient content (N, P, K, Ca, Mg, and Zn) in leaves of high and low-yielding subpopulations of cowpea.

Nutrients	High-yielding population (93)		Low-yielding population (99)	
	Mean	SD	Mean	SD
N (g/kg)	44.39	6.71	31.60	8.46
P (g/kg)	11.85	3.37	6.65	2.24
K (g/kg)	33.06	6.70	28.98	6.25
Ca (g/kg)	90.93	17.09	84.76	10.71
Mg (g/kg)	7.40	2.37	5.66	2.38
Zn ((g/kg)	0.06	0.01	0.05	0.01
Yield (kg/ha)	1094.45	75.49	802.23	93.15

**Table 2:** DRIS norms are represented by the average, standard deviation (SD), and coefficient of variation (CV) of the dual ratio between nutrients in high yield subpopulation.

DRIS ratios	Mean	SD	CV	*P* value (Shapiro–Wilk test)
N/P	5.08	0.8	15.74	0.07
K/N	0.76	0.11	14.47	0.32
Ca/N	2.11	0.37	17.53	0.08
Mg/N	0.17	0.03	17.64	0.09
Zn/N	0.00	0.00	10.00	0.61
K/P	2.99	0.53	17.72	0.06
Ca/P	8.2	1.46	17.80	0.21
Mg/P	0.71	0.12	16.90	0.44
Zn/P	0.01	0.00	0.00	0.19
Ca/K	2.83	0.56	19.78	0.32
Mg/K	0.23	0.03	13.04	0.07
K/Zn	608.92	107.32	17.62	0.09
Ca/Mg	14.53	2.67	18.37	0.12
Ca/Zn	1682.01	333.23	19.81	0.31
Mg/Zn	132.25	24.13	18.24	0.08

### DRIS Indexes and nutrient requirements for Cowpea

**Figure 2:**
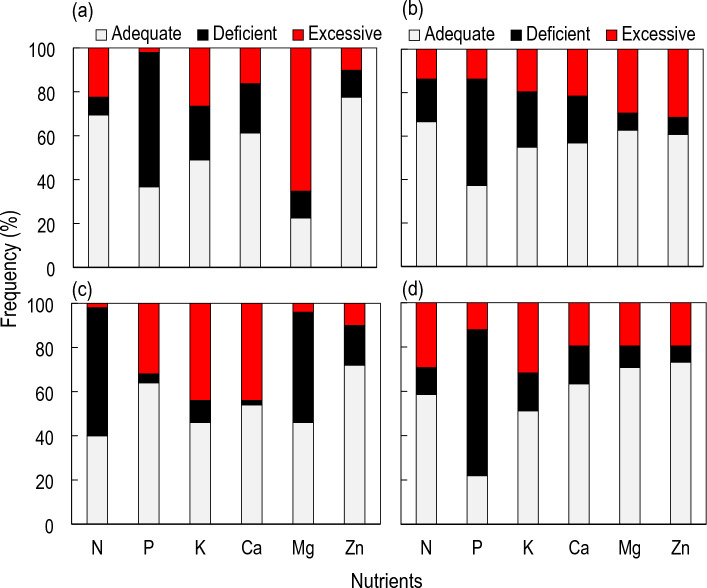
Nutritional status of the cowpea plant population at Dassa-Zoume (**a**), Glazoue (**b**), Ketou (**c**), and Ouesse (**d**) as indicated by the DRIS assessment of cowpea leaves.

Based on DRIS indexes, P was the most required element among nutrients at Glazoue, Dassa-Zoume, and Ouesse while N was the most required nutrient at Ketou (Table 3). In addition to P and N, Mg and Zn were also important nutrients at Dassa-Zoumè, Ketou, and Ouesse. It is worth noting that cowpea was not often fertilized with these nutrients. However, based on the DRIS nutritional assessment, the order of nutrient deficiency in the cowpea plant population was as follows: P > K > Ca > Zn > N > Mg; P > K > Ca > N > Zn > Mg; N > Mg > Zn > K > P > Ca; P > Ca > K > N > Mg > Zn, at Dassa-Zoume, Glazoue, Ketou, and Ouesse, respectively (Fig. 2). Although, on a municipal scale, K and Ca were not deficient (Table 3), there are deficiencies in K and Ca in some locations where leaf tissues were sampled (Fig. 2).

**Table 3:** DRIS indexes and order of nutrient requirement.

Location	DRIS index						Order of nutrient requirement
	N	P	K	Ca	Mg	Zn	
Glazoue	0.00	− 2.62	0.45	1.15	0.22	0.79	P > N > Mg > K > Zn > Ca
Dassa-Zoume	− 0.21	− 1.44	0.21	0.41	1.35	− 0.32	P > Zn > N > K > Ca > Mg
Ketou	− 1.65	0.00	0.92	1.42	− 0.62	− 0.07	N > Mg > Zn > P > K > Ca
Ouesse	0.55	− 3.07	0.91	1.10	− 0.18	0.68	P > Mg > N > Zn > K > Ca

### Spatial interpolation of nutrient DRIS indexes

**Figure 3:**
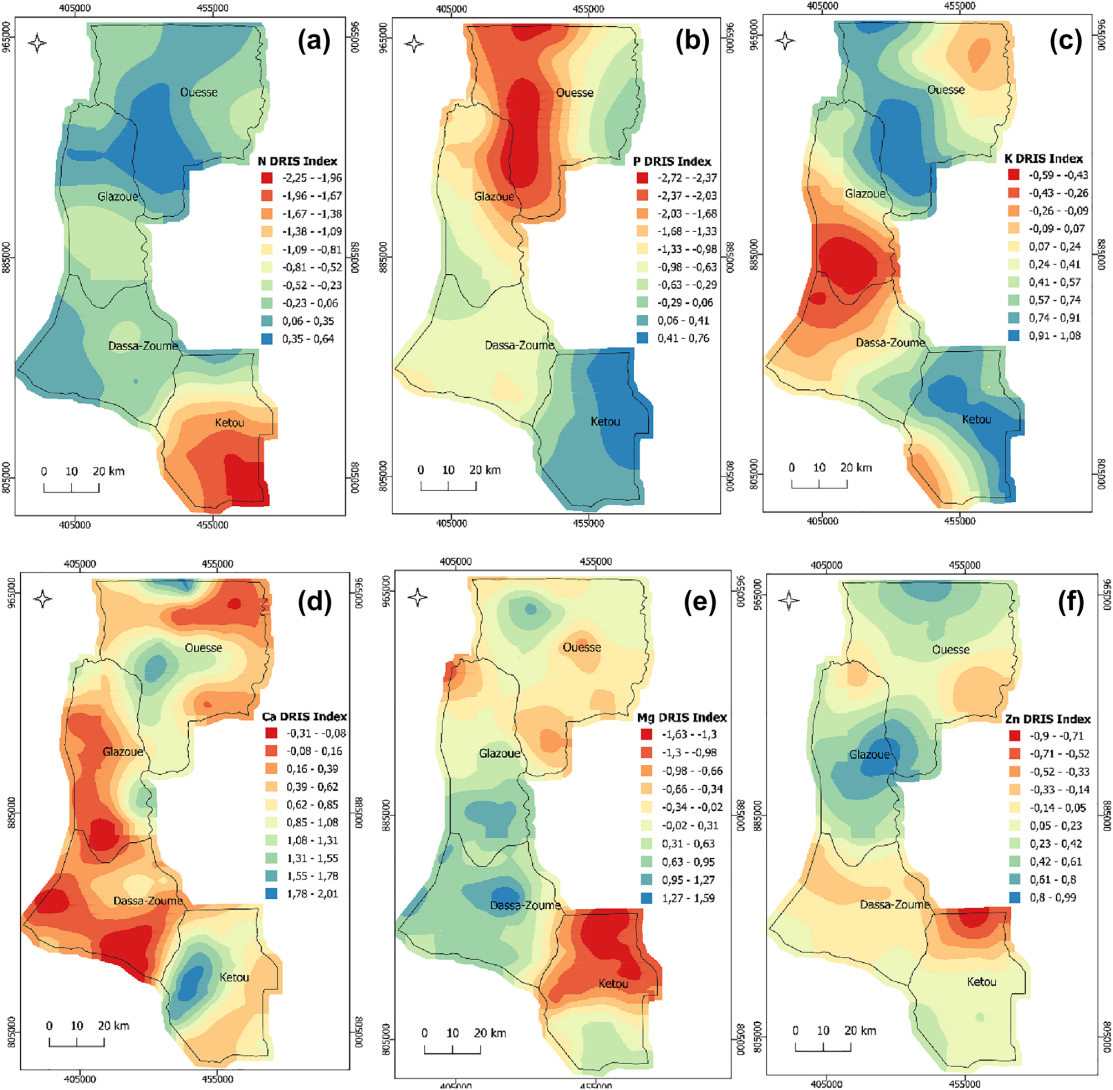
Spatial distribution maps of DRIS index of N (**a**), P (**b**), K (**c**), Ca (**d**), Mg (**e**), Zn (**f**).

All DRIS indexes presented moderate spatial variability (Table 4). The analysis of spatial distribution maps of DRIS indexes showed the locations where each nutrient would be deficient with negative indexes (Fig. 3), although, at the regional scale, K and Ca were not deficient in all municipalities, Mg and Zn were not deficient in Glazoue only (Table 3). Within the same municipality, nutrient status strongly varied from one geographic location to another (Fig. 3). The south zone of Ketou was strongly deficient in N. This deficiency decreased from south to north Ketou. In other municipalities, N deficiency was low with some places where N was excessive (Fig. 3a). The distribution map of DRIS indexes of P indicated the large zone where P was deficient with an extreme deficiency observed from north of Glazoue to the northeast of Ouesse (Fig. 3b). The distribution map of DRIS indexes of K showed that this nutrient was deficient from the north of Dassa-Zoume to the center of Glazoue (Fig. 3c). However, K and Ca DRIS index values were positive in all municipalities (Table 3). Ca was generally not deficient in the study area although there are small zones of deficiency was depicted in almost all municipalities (Fig. 3d). On basis of Mg DRIS values, the deficiencies were more pronounced in the north of Ketou, and moderate deficiencies were observed in Ouesse and north of Glazoue (Fig. 3e). Likewise, Zn was strongly deficient in the north of Ketou and moderately deficient in Dassa-Zoume and northwest of Ouesse (Fig. 3f).

**Table 4:** Parameters of the fitted variograms for the DRIS Indexes, NBIm, and relative cowpea yield.

Variables	Model	C_0_	C_0_ + C	SDI	R^2^
Index N	Gaussian	8.5	26.7	68.16	64.2
Index P	Exponential	13.7	35.5	61.41	46.5
Index K	Spherical	46.4	108.1	57.08	56.4
Index Ca	Spherical	15.6	54.7	71.48	71.7
Index Mg	Gaussian	25.8	64.6	60.06	65.8
Index Zn	Gaussian	23	64.4	64.29	52.3
NBIm	Spherical	18.2	46.2	60.61	59.7
Relative yield	Gaussian	26.5	98	72.96	57.4

C_0_ = Nugget; C_0_ + C = Sill; SDI = Spatial dependence index (C/C_0_ + C)*100 and R^2^ = coefficient of determination of the variogram model.

### Spatial variability of mean nutrient balance index (NBIm) and relative cowpea yield

**Figure 4:**
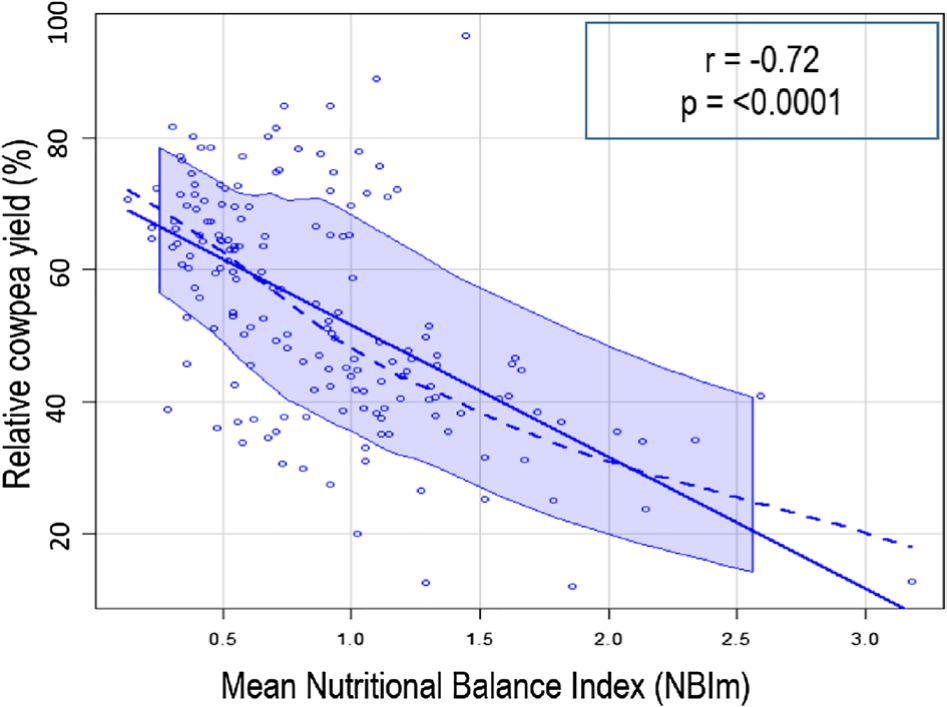
Relationship between NBIm and relative cowpea yield.

**Figure 5:**
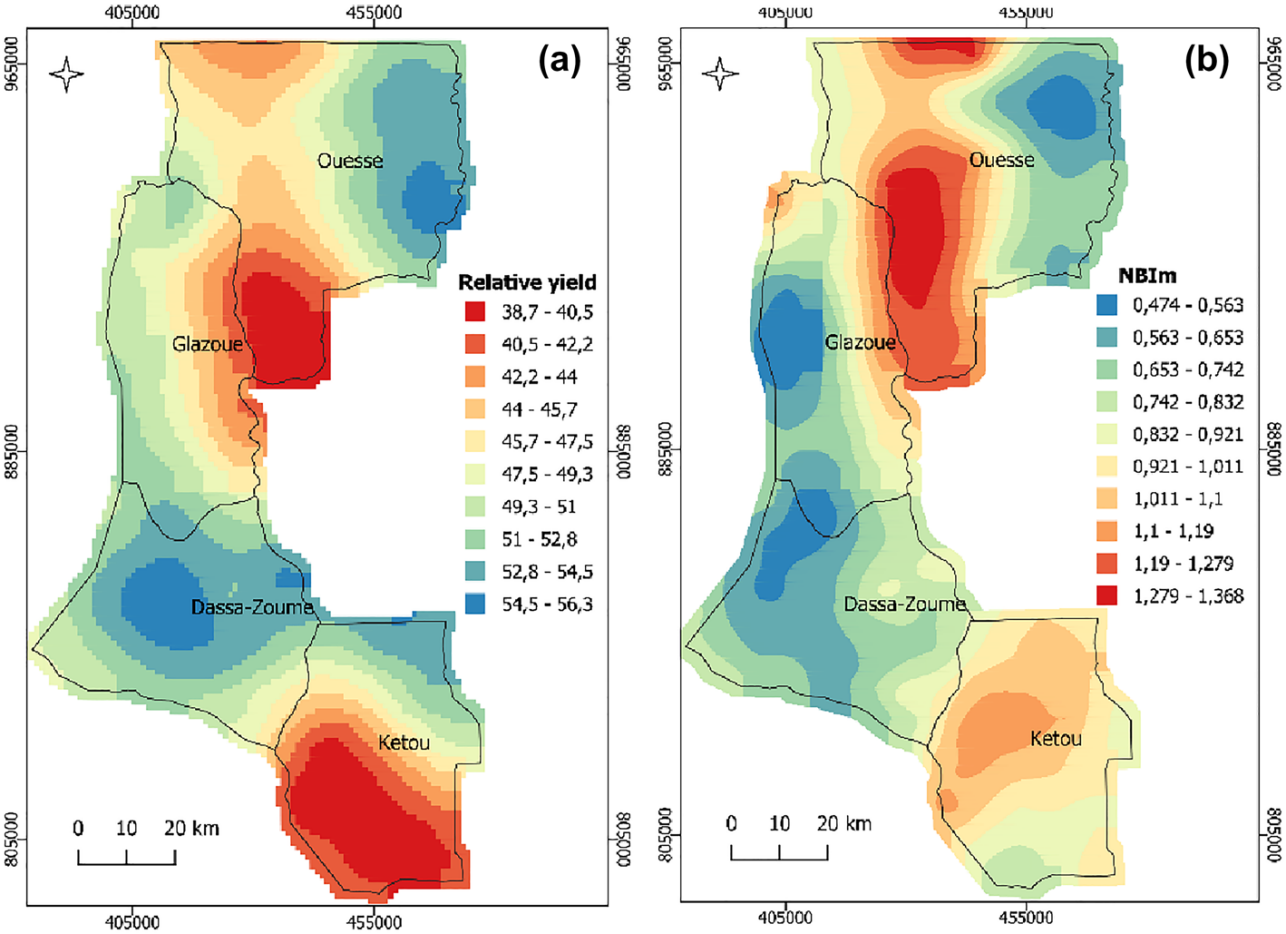
Spatial distribution maps of relative cowpea yield (**a**) and NBIm (**b**).

The analysis of dispersion between relative cowpea yield and NBIm value indicated a negative linear correlation, since for the range of low NBIm, high relative cowpea yield was obtained (Fig. 4). The relative cowpea yield map has around 54% of its total area under low yield, with relative cowpea yield values ranging from 38.7 to 49.3% (Fig. 5a). On the NBIm map, these low yield zones have high NBIm, which ranged from 0.92 to 1.37, while zones with relatively high cowpea yield (> 52%) have low NBIm, which was less than 0.65 (Fig. 5b). The relative cowpea yield and NBIm presented moderate spatial variability (Table 4).

The NBIm map, effectively indicates the spatial distribution of cowpea productivity as the nutritional status of the plants is the major limitation to production. From south to center Ketou, relative cowpea yield was very low while, NBIm was not relatively high, which shows a weak relationship between NBIm and relative yield in this area (Fig. 5).

The value of NBIm indicated the level of nutritional imbalance in the cowpea plant without specifying the nutrient and whether it is excessive or deficient. However, the combined analysis of spatial distribution maps of NBIm and each nutrient DRIS index indicated the details and precisions about the use of spatial distribution maps of NBIm. As such, nutrient imbalances observed in south of Ketou (Fig. 5b) were mainly explained by the severe deficiency of N (Fig. 3a). In addition, N DRIS indexes were negative and its absolute values were higher than NBIm. Therefore, N application in south Ketou will get positive potential responses. In contrast, in the north of Ketou, the nutrient imbalances were instead explained by severe deficiency of Mg with negative DRIS indexes and absolute values higher than NBIm (Fig. 3e). Such results indicated that Mg application in north Ketou would result in positive potential responses. The severe nutrient imbalances observed from north of Glazoue to north of Ouesse (Fig. 5b) were mainly explained by severe deficiency of P (Fig. 3b). In this zone, P application would result in positive potential responses. However, in the east of Ouesse, the P application will get null potential responses. Combined analysis of distribution maps of NBIm and K DRIS index indicated that the northwest of Dassa-Zoume and south of Glazoue have nutrient imbalance mainly explained by K deficiency (Figs. 3c and 5b). Based on the K DRIS index and NBIm values, the K application would result in positive potential responses in these zones.

## Discussion

Our study first revealed that the integration of geostatistical tools as a means for continuous diagnosis of crop nutritional status using the DRIS approach is efficient to evaluate soil fertility. Secondly, DRIS norms established indicated that the proper relationship between N and P in cowpea leaf to obtain high yield was N/P = 5.08, and other nutrients such as K, Ca, Mg, and Zn were required in cowpea cultivation. Phosphorus is critical to cowpea yield because of its multiple effects on plant nutrition^42^. P increases cowpea yields^2^ but also nodulation^43,44^ and thus N fixation. Therefore, the average N/P nutritional relationship would be considered very important in cowpea nutritional evaluations. Unfortunately, in many farmers' fields, fertilizers are not used for cowpea cultivation^2^. Although the importance of P fertilization in cowpea cultivation has been studied, the results of this study revealed that other leaf nutrients levels such as K and Ca were more important than P in establishing nutrient balance. In addition, to balance nutrients, cowpea requires nearly the same leaf content of Mg and P. These nutrients would only be available to the cowpea crop through fertilization or soil content. However, in many places in Sub-Saharan Africa, soils are severely deficient in nutrients, including N, P and K, Ca and Mg, and the micronutrient Zn^45,46^. These nutrient deficiencies could explain the low yields of cowpea observed in farmer’s fields. Based on the DRIS index averages on the municipality scale, P, N, Mg, and Zn were the nutrients deficient in many locations, which suggests fertilization with such nutrients. This is important since in areas under cowpea cultivation the use of P, N, Mg, and Zn fertilizers is rarely practiced^2^, which can lead to a nutrient misbalance inhibiting cowpea vegetative growth and the subsequent productive performance. Nonetheless, in the municipality where P, N, Mg, and Zn were deficient, based on DRIS nutritional assessment on leaf samples, there are some locations where these nutrients were adequate or excessive. In addition, K and Ca were deficient in some locations, while their DRIS index averages did not show deficiencies. These results showed that the interpretation of DRIS index averages could not provide efficient output to optimize farming techniques and application of chemical fertilizer nutrients. The major challenge in soil fertility management is to stabilize the required amount of nutrients based on soil type, crop need, and environment^1^. Therefore, the recommendations generated on leaves samples drawn from the grid sampling system cannot be generalized to the entire area.

This study shows the strong linear correlation between NBIm and relative cowpea yield. Our results are consistent with da Silva et al.^11^ who reported the correlation between the Nutritional Balance Index (NBI) and the yield of the coffee plant. Likewise, de Morais et al.^36^ observed a correlation between whole plant dry matter (DM) of *Eucalyptus spp* and NBIm obtained using the DRIS. The similarity observed between spatial distribution maps of relative cowpea yield and those of NBIm in this study shows the greater efficiency of the DRIS system in diagnosing cowpea nutritional status. This result suggests that spatial distribution maps of NBIm could be used as an efficient tool in fertilization programs. Indeed, the greater the relationship between NBIm and yield improves the diagnostic system response, to point out the nutritional status of plants^37,47^. From south to the center of Ketou, the relatively low yield, and NBIm observed indicate that other factors than nutritionally limited cowpea productivity. Therefore, both spatial destruction maps of relative yield and NBIm improve nutritional status appreciation and make easy the identification of areas where it is expected that other factors were limiting cowpea productivity. NBIm can be a useful tool to indicate the nutritional status of the plant because, the higher the NBIm, the greater the nutritional imbalance^12^. However, it does not discriminate against the nutrient that would be limiting the yield. Our study suggests that interpretations of spatial distribution maps of NBIm with each nutrient DRIS index may be effective to indicate the areas where the nutrient application will get potential positive or no responses for cowpea cultivation. These findings are consistent with Ribeiro et al.^25^ who revealed that in the spatial variability of nutrient indexes, it is possible to see very restricted points of deficiency and excess in the sampling area of plant tissue. However, in this single interpretation of spatial variability of the nutrient index, it is important to highlight that the regions with suitable nutritional status show values of nutrient index near zero^12,13,25,48^. In practice, the probability of having zero values for the nutrient index is small. Thus, NBIm was used as a value that reflects the average of the deviations of each dual ratio of nutrients relative to the reference value^41^. The combined interpretation of spatial variability of NBIm and nutrient index allows appreciation of spatial variability of nutrient application potential responses.

## Conclusion

Continuous assessment of cowpea crops’ nutritional status by geostatistic tool and Diagnosis and Recommendation Integrated System was efficient and well reflected the current status soil fertility management. Phosphorus, nitrogen, magnesium, and zinc were the most required nutrients. The correlation between the mean of the nutritional balance index and relative yield was significantly strong, suggesting that for the studied cowpea area, the nutritional factor is the largest yield limiting factor. The use of a geostatistics tool combined with the Diagnosis and Recommendation Integrated System index, and mean nutrient balance index resulted in an improved understanding of the influence of nutritional, and non-nutritional variables on cowpea yield variability.

## Data Availability

The datasets used and/or analyzed during the current study are available from the corresponding author upon reasonable request.
